# Cultural evolution, social ratcheting and the evolution of human division of labour

**DOI:** 10.1098/rstb.2023.0277

**Published:** 2025-03-20

**Authors:** Lucio Vinicius, Leonardo Rizzo, Federico Battiston, Andrea Bamberg Migliano

**Affiliations:** ^1^ Human Evolutionary Ecology Group, University of Zurich, Zürich 8057, Switzerland; ^2^ Department of Network and Data Science, Central European University, Vienna 1100, Austria; ^3^ Department of Decision Sciences, Bocconi University, Milan 20136, Italy

**Keywords:** social ratcheting, cumulative culture, individual cognition, cultural transmission

## Abstract

While ecological specialization, social differentiation and division of labour are found in many species, extensive and irreversible interdependence among culturally specialized producers is a characteristic feature of humans. By extending the concept of cultural ratcheting (or the evolution of cultural products of such complexity that they become very unlikely to be recreated from scratch by naive individuals), we present simulation models showing how cumulative cultural evolution may have engendered a parallel process of ‘social ratcheting’ or the origin of culturally differentiated and irreversible interdependent individuals and groups. We provide evidence that the evolution of cultural division of labour in humans may have been associated with social network structures splitting the cognitive costs of cultural production across differentiated specialists, significantly reducing the burden of cultural learning on individual cognition and memory. While previous models often assumed agents with unlimited memories, we show that limiting individual memories to a fraction of available cultural repertoires has a noticeable accelerating effect on both cultural evolution and social differentiation among producers. We conclude that cultural and social ratcheting may have been two linked outcomes of cultural evolution in the hominin lineage.

This article is part of the theme issue ‘Division of labour as key driver of social evolution’.

## 1. Introduction

Products of cumulative cultural evolution may reach a level of complexity beyond which they become more unlikely to be recreated from scratch by naive individuals [1–3]. Cultural products that can only be individually acquired through social transmission define a cognitive threshold known as cultural ratcheting [4]. A related feature of cumulative cultural evolution is social division of labour [5]. For example, while an extreme example of cumulative culture such as a space station consists of a large number of interconnected components, it also requires the existence of space engineers alongside many other specialized individuals. We proposed the term ‘social ratcheting’ to describe the point beyond which cultural specialists can no longer recreate cultural products on their own and become interdependent agents within division of labour systems [6].

In comparison with the vast literature on human cumulative culture, the evolution of human division of labour has remained relatively understudied [7,8]. This may be partially due to previous research not always clearly delimiting the boundaries among the related but distinct processes of specialization, differentiation among specialists and finally division of labour among differentiated and interdependent specialists. For example, Smolla & Akçay [9] have modelled the effect of cultural transmission on network structure and individual skill sets, concluding that selection for skill specialization was associated with increased network density and more proficient individuals. However, their model also predicted that interconnected specialists would converge towards the same skill, leading to the loss of cultural differentiation and diversity at the population level, conclusions at odds with what is observed in human societies. The reason seems to be that their model may be more appropriate to simulate the evolution of ecological specialists [10], where there is no division of labour or interdependence within populations, in contrast to human societies characterized by differentiated cultural specialists, and high cultural diversity at the population level.

Apart from humans, another frequently discussed instance of social division of labour is eusociality and caste differentiation in some arthropods and mammals, which can be satisfactorily explained by a combination of kin selection (reduced within-group conflict among close relatives owing to haplodiploidy in Hymenoptera; or high relatedness in termites, aphids and mole rats) and ecological factors (living in defensible nests, exploring abundant and concentrated resources; [11–13]). However, major aspects of eusociality are absent from humans: non-human apes do not exhibit eusociality (and neither did our shared common ancestor), *Homo sapiens* since its origins was likely to show lower levels of within-group relatedness than other apes, and we do not observe a distinction between reproductive and non-reproductive castes in humans [14,15].

The main factor preventing a straightforward comparison between humans and eusocial species is that human division of labour and interdependence are culturally rather than genetically established. Some field studies have suggested that culture can promote differentiation of behaviours and skills in apes [16,17]. At the population level, nut-cracking in chimpanzees has been found only in a few populations in Central Africa, despite the availability of nuts and stones in other locations [18,19]. Social learning studies [20,21], field observations [22,23] and correlations between genetic interconnectivity and tool sharing [24] have strongly suggested that nut-cracking is culturally transmitted between populations. At the individual level, anecdotal reports range from describing certain complex technologies as customary (nut-cracking shared among virtually all individuals in Bossou; [25]), common (ant fishing being performed by over half of adults in Kasekela, Gombe; [26]) or rare (nut-cracking using a wedge stone in Bossou; [27]). However, there is no evidence of extensive exchange of products or skills between adults, skill complementarity in a single task (with a single multi-step task being performed by individuals specialized in different steps) or exchange of products requiring distinct skills, and so far there is no systematic summary of skill distribution at an individual level in chimpanzees. Overall, although cultural specialization and differentiation may occur, evidence of division of labour and interdependence is scant even in a culturally rich and diverse species such as chimpanzees.

In contrast, division of labour is a universal feature of current human societies, including the few extant hunter–gatherers, and is likely to have evolved in the deep past of *H. sapiens* [6,8]. Hunter–gatherers from Central Africa show a variety of specialists such as public speakers, spirit guardians, individuals in charge of communicating with external groups, song composers and ritual runners [28], and even sub-specialists, including elephant hunters as opposed to regular hunters, and healers catering specifically for infant health, sorcery, family issues or exorcism, among others [29]. The archaeological record provides evidence of trade and exchange of ostrich eggshell beads from at least 33 ka in southern Africa [30], remarkably similar to the *hxaro* gift system which circulates tools and ornaments among households and groups of extant San hunter–gatherers from Namibia [31]. Since *hxaro* beads are now only produced by women, the fossil beads hint at the possibility of sex division of labour at least since the Late Stone Age.

All this evidence suggests that ecological specialization and cultural differentiation seem insufficient to capture the key feature of human social ratcheting, namely the evolution of irreversible interdependence among culturally differentiated individuals. In addition, division of labour in eusocial species is as a rule associated with high relatedness among cooperators and explained as an outcome of indirect fitness effects [32]. For those reasons, we believe that more appropriate biological analogies to the evolution of cultural division of labour in humans seem to be cases where cooperation was more likely to have evolved among unrelated individuals, such as in the symbiotic associations behind the first eukaryotic cells [33] or the evolution of interdependent mating types in sexual species [34]. Such instances of biological aggregation [35] were later generalized by the concept of major transitions in evolution [36], or unique evolutionary processes that led to the emergence of new entities resulting from specialization, differentiation, division of labour and irreversible interdependence among cooperative and complementary units. The logic of major transitions implies that new entities evolve owing to selective pressures at the level of their ancestral components: kin selection in the case of multicellular organisms or eusociality, or cooperation among unrelated individuals in the case of sexual reproduction or symbiosis [37].

Human societies were interpreted by Maynard Smith & Szathmáry [37] as the most recent of the major evolutionary transitions, with cooperation and interdependence both among related and and among unrelated individuals, interconnected by language as a new form of transmission of biological information. However, it is widely recognized that culture as a whole has played a more general role than language in the origins of human division of labour [38,39]. Rather than language alone, it may be more appropriate to refer to cultural accumulation and transmission as the key drivers of human social organization, both to highlight the overlap between the concepts of major transitions and dual inheritance systems [40,41], and to stress that language itself is a product of gene–culture coevolution [42].

A few attempts were made at demonstrating the links between cultural transmission and division of labour in humans. For example, Henrich & Boyd [7] examined the effect of variation in cultural learning strategies on division of labour, but they assumed that groups were already sufficiently isolated to create opportunities for specialization. As pointed out by Nakahashi & Feldman [5], their analytical model does not address how differentiation and stable division of labour would emerge within a group of individuals originally with the same skills, a conspicuous feature of human societies. However, Nakahashi & Feldman’s own equations assume that cultural specialists are limited by the number of available resources (in their model, only two) and mostly apply to gender division of labour. Although the two studies have the merit of establishing a link between cultural evolution and division of labour, they do not consider more generally the longer-term interactions among cumulative cultural evolution, social structure and resource exploitation in the hominin lineage. For example, stepwise evolution of new lithic and non-lithic technologies was likely to have occurred hand-in-hand with an expansion of the range of available resources to hominins [43], and this may have increased opportunities for increasing cultural division of labour.

We have previously argued that a growing dependence of earlier hominins on lithic technologies [6] led to both cultural ratcheting (the irreversible dependence on social learning for the acquisition of cultural skills) and social ratcheting (the irreversible interdependence among cultural specialists). Therefore, cumulative culture may be seen as the factor favouring selection for increasing division of labour in hominins to a point where benefits to specialists with complementary cultural skills resulted in irreversible interdependence. A possible advantage of cultural specialization at the individual level is that storage and learning of cumulative culture may be spread across networks [38]. Network memories [44,45] may significantly reduce cognitive demands on individual memory and learning [46]. However, previous models have mostly postulated agents with unlimited memories, an unrealistic assumption given the potential cognitive load of cultural repertoires growing in size and complexity [47]. The potential effects of cognitive overloads on human memory and learning on division of labour have not yet been investigated in detail.

Another feature of human division of labour is that it relates to cooperation at two distinct levels: distribution and production. Food sharing in hunter–gatherers [48,49], gift systems [50] and trade [51] may have evolved as a result of general advantages of product distribution among groups and individuals. In contrast, cooperation in production (sharing of complementary knowledge or tools) directly involves only producers. For this reason, cooperative processes behind production or distribution may affect social structures and exchanges in different ways. For example, studies showing the positive effects of partial connectivity in experimental networks [46] and of multilevel sociality of extant hunter–gatherers [44] on recombination, innovation and the evolution of cultural complexity have mostly focused on the sharing of knowledge among producers.

In the following, we present agent-based models to investigate whether cumulative cultural evolution promotes not only cultural ratcheting but also social ratcheting or division of labour in the form of culturally specialized, differentiated and interdependent individuals and groups. Agent-based models have been frequently applied to systems where behavioural rules can be defined at the individual level, but outcomes on a social and evolutionary scale depend on various interactions among multiple agents, between agents and physical or cultural environments, and over many generations. Agent-based modelling is suited to evolutionary studies since agents can be designed with variable behavioural patterns, life histories, mutation rates among other traits. They are particularly appropriate to the study of cultural evolution as they can easily incorporate behaviours such as social learning and innovation, as well as the accumulation, loss, gradual improvement and recombination of cultural traits. For example, agent-based models by Derex & colleagues [52] investigated the effects of population structure on cumulative cultural evolution and demonstrated that more complex traits tend to evolve in populations characterized by intermediate levels of social interconnectivity, as they provide a balance of cultural loss and diversity. Migliano *et al*. [44] have also used agent-based simulations to demonstrate that the multilevel social structures found in extant hunter–gatherers exhibit features of partial connectivity and accelerate cumulative cultural evolution. Cantor *et al.* [53] supplemented the previous analyses by revealing that various network structures engender partial connectivity, and also that variation in social network structure may have distinct effects on rates of creation and diffusion of complex cultural traits. Although the previous works have furthered our understanding of cumulative cultural evolution, agent-based modelling has not yet been applied to the problem of cultural division of labour.

In the following, we start by simulating cultural evolution, including both incremental improvements along differentiated lineages and crossovers between lineages generating technological leaps in production. We propose various measures of specialization, differentiation, division of labour and interdependence to assess social ratcheting. Our agent-based models address how two factors, namely limited versus unlimited agent memory, and presence versus absence of cultural transmission, affect rates of cultural and social ratcheting.

## 2. Methods

### (a) Simulation parameters

**Figure 1.**
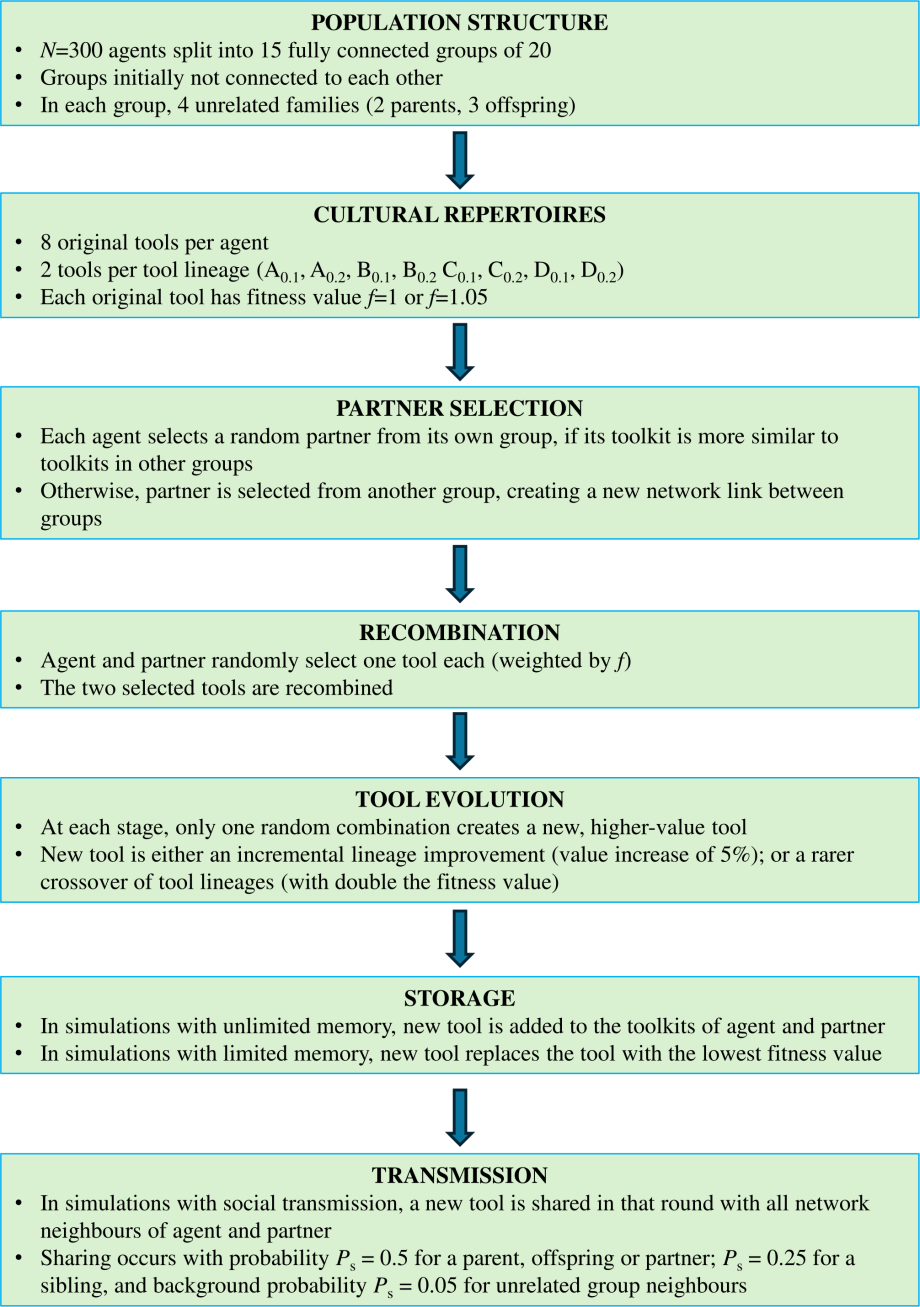
Flowchart of agent-based simulations.

In each simulation, an initial population of *n* = 300 individuals was divided into *M* = 15 groups (figure 1). Each group was structured as a fully connected network, and its 20 members were split into four unrelated families (two parents and three offspring each). We then simulated a process of cultural evolution closely following previous work [44,46]. Each individual was initially endowed with a set of eight tools from four cultural lineages (two tools per lineage). Each tool was ascribed a baseline fitness value (1 or 1.05).

### (b) Partner selection

At every simulation round, each agent estimates its toolkit distance to the other 299 agents as the Jaccard distance *J* = 1−number of common tools/total number of tools . The agent then selects a partner either from its own group or from another, depending on the parameter *P* = $\frac{Db\;-\;Dw}{2}$ , with *D*_b_ as the mean toolkit distance to the 280 agents from other groups and *D*_w_ as the mean toolkit distance to the other 19 agents within its group. When an agent is more similar to agents in its own group (*p* > 0), a random partner is selected from another group with probability *P*; otherwise (*p* ≤ 0), a random partner is selected from the same group. As seen, *P* is dynamic and changes as a function of toolkit differences at each step of the simulation.

### (c) Tool creation and crossovers

After partners are selected, each agent in a dyad randomly selects one tool from their toolkit with probability proportional to tool fitness. Among all possible tool combinations, a randomly chosen one results in the creation of a new and superior tool with higher fitness value. If the selected tools do not generate a new tool, the simulation moves on to another agent until all 300 agents select a partner and a full round is complete. If a new tool is discovered, both agents add the new tool to their toolkit. Simulations end after 150 rounds.

*L*_m.n_ denotes a tool at the stage *m* and level *n* in lineage *L*. Each technological stage has three incremental levels: *L*_m.1_, *L*_m.2_, *L*_m.3_. For example, tool *A*_3.2_ is at the second incremental level of the third stage. Technological evolution has two modes: incremental increases between levels or technological transitions between stages. To reach the incremental level *n* at the stage *m*, agents must combine two randomly selected tools of level *L*_m.(*n*−1)_ and *L*_m.(*n*−2)_ from the same tool lineage. The agent is classified as belonging to the tool lineage of its most evolved tool. Therefore, agents can shift across the four lineages during a simulation. If at some point an agent has more than one most evolved tool from different tool lineages, it is randomly and momentarily assigned to one of them. To achieve a crossover *m* + 1, agents must recombine two randomly selected *L*_m.3_ tools from different cultural linages. When a crossover occurs, the newly created tool is placed in the same lineage as the tool contributed by the first agent in the dyad. For example, if an agent with an *A*_1.3_ tool selects a partner with a *B*_1.3_ tool, successful recombination generates an A_2.1_ tool; if the positions in the dyad were inverted, the outcome would be a *B*_2.1_ tool. Incremental advances lead to a 5% increase in fitness compared with the previous level, while crossovers double the fitness value of the discovered tool.

A newly created tool is added to the toolkit of both agent and partner and is shared in that round (in scenarios with social transmission) with all their network neighbours with probability *P*_s_ = 0.5 for a parent, offspring or partner, *P*_s_ = 0.25 for a sibling, and background probability *P*_s_ = 0.05 for unrelated group neighbours. *P*_s_ values are meant to broadly indicate that sharing correlates with relatedness.

### (d) Simulation scenarios

We have run the simulations under four distinct scenarios based on two variables, namely agent memory and social transmission. We treated memory (unlimited versus limited) and social transmission of tools to neighbours (present versus absent) as independent factors, resulting in four simulation scenarios: (i) unlimited memory, no transmission; (ii) limited memory, no social transmission; (iii) unlimited memory, social transmission; and (iv) limited memory, social transmission. In simulations where agents have limited memory (eight items), a new tool (either created or transmitted from another agent) replaces the tool with the lowest fitness value. In simulations with social transmission, *P*_s_ probabilities above are applied. We ran simulations 50 times for each scenario and report mean and standard errors in the figures. In the electronic supplementary material, we explore two parameters in more detail, namely population size (both group size and number of groups) and agent memory size (ranging from eight to unlimited tools).

### (e) Entropy estimator

Information or Shannon entropy was calculated as


$$H\left(X\right):=-\sum_{x\in \left\{A,B,C,D\right\}}p\left(x\right)\log p\left(x\right),$$

where x can take four values of the cultural lines. The probabilities $p\left(x\right)$ rendering unweighted entropies were calculated as


$$p\left(A\right)=\frac{\text{number}\;\text{of}\;\text{tools}\;\text{in}\;A}{\text{total number of tools}}$$

Entropies weighted by the fitness values of tools were calculated as


$$p{\left(A\right)}_{\text{fit}}=\frac{\sum_{i\in A}fitness\left(i\right)}{\sum_{\forall i}fitness\left(i\right)},$$

where fitness(i) is the function that assigns a fitness value to tool *i*.

## 3. Results

### (a) Memory limitation and social transmission accelerate cultural evolution

**Figure 2.**
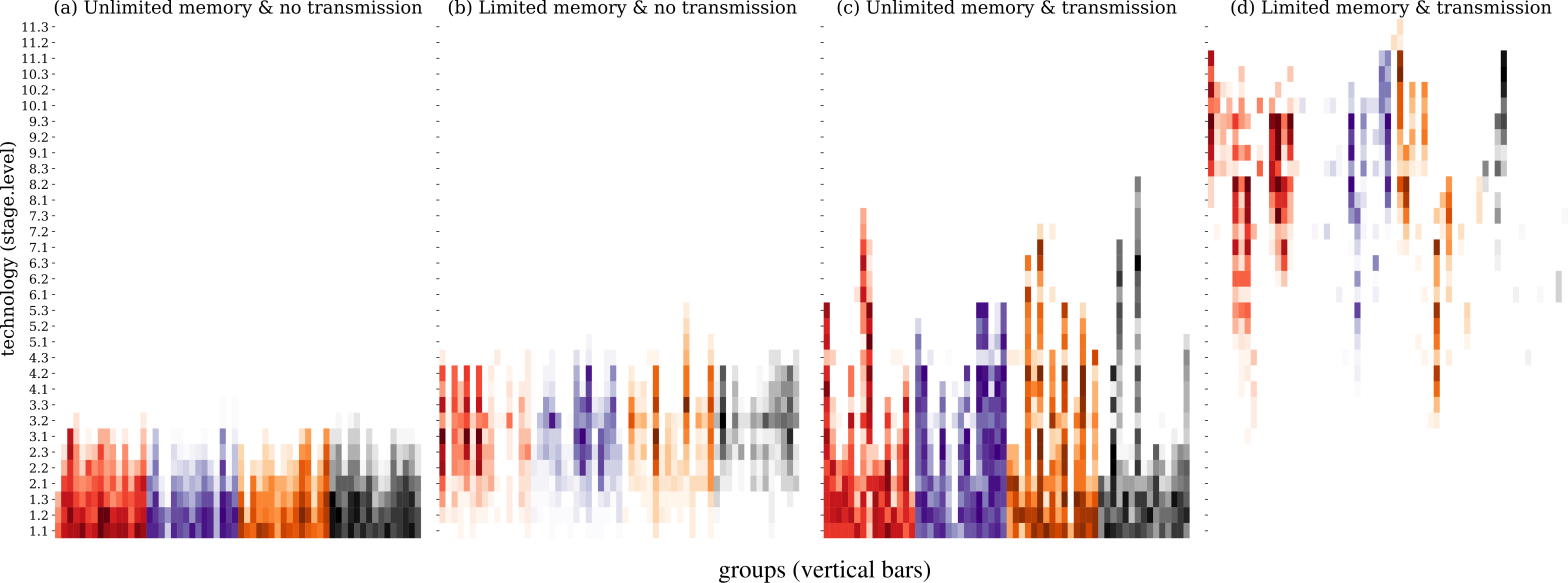
Cultural evolution simulations under four scenarios: (*a*) agents with unlimited memory and no social transmission, (*b*) limited memory (eight items) and no social transmission, (*c*) unlimited memory and social transmission, and (*d*) limited memory and social transmission. The *y*-axis represents evolved technological stages and levels. Vertical bars on the *x*-axis correspond to the 15 groups in each of the four scenarios. Colours represent the four evolving technological lineages (*a–d*). Colour shades in each vertical bar represent the fraction of agents storing a given tool.

Our simulations revealed noticeable differences in tool evolution across the four scenarios. Technological evolution was slowest when agents had unlimited memories, and pairs could not transmit newly created tools to their network neighbours (figure 2a). After 150 rounds, the maximum number of technological leaps (crossovers between lineages) was 2, with the resulting tools reaching stage 3. The introduction of social transmission had a pronounced effect, with tools evolving to stage 8 (figure 2c). This confirms that the partial spread of innovations primarily to family members, and less likely to other unrelated group members, favours cultural accumulation [54].

We also identified an independent effect of memory limitation. In the scenarios without social transmission, a limitation of agent memory to eight items also facilitates technological leaps, and tools evolve to stage 5 (figure 2b). The finding that a limited memory that reduces the size of individual toolkits can accelerate tool evolution seems counterintuitive. However, it suggests a trade-off between individual memory and collective tool repertoire, confirmed by additional simulations showing that increases in maximum item memory from eight to unlimited items gradually slow down tool evolution (see electronic supplementary material, figures S1 and S2 ). Finally, the combination of memory limitation and social transmission had an even stronger effect, with cultural innovations reaching stage 12 in some populations (figure 2d). The trade-off between memory size and tool complexity is observed under different population sizes, which we explored by varying both group size and group number (electronic supplementary material, figures S3–S5). In summary, the combination of limited agent toolkits and partial spread of innovations seems to accelerate both the rates of incremental technological evolution and occurrence of rarer technological leaps.

### (b) Memory limitation and social transmission increase agent specialization and differentiation

Next, we evaluated possible effects of memory and transmission on cultural specialization and differentiation of agents. In principle, unlimited agent memories should allow the accumulation of newly created tools and skills without cognitive costs, and for this reason might be expected to favour the evolution of cultural generalists rather than specialists. On the other hand, general transmission of new findings to all nodes in a network tends to reduce diversity in individual toolkits [46]. To assess levels of toolkit specialization among individuals, we selected information entropy as a metric estimating the probability of a given toolkit among all possible tool combinations when tools are classified into one of the four available technological lineages. A high-entropy toolkit contains a relatively homogeneous distribution of tools across the four lineages, identifying an agent with low cultural specialization. Low entropy corresponds to agents concentrating tools from fewer or a single lineage and hence highly specialized. If all tools in a toolkit come from one lineage alone, entropy = 0 and the agent is maximally specialized. We estimated agent entropy both weighting and not weighting tools by their fitness values. Weighting was included to account for the fact that high-value tools are more likely to be selected in recombination events, and to prevent artificial inflation of entropy in unlimited memory scenarios where no tools are discarded.

**Figure 3.**
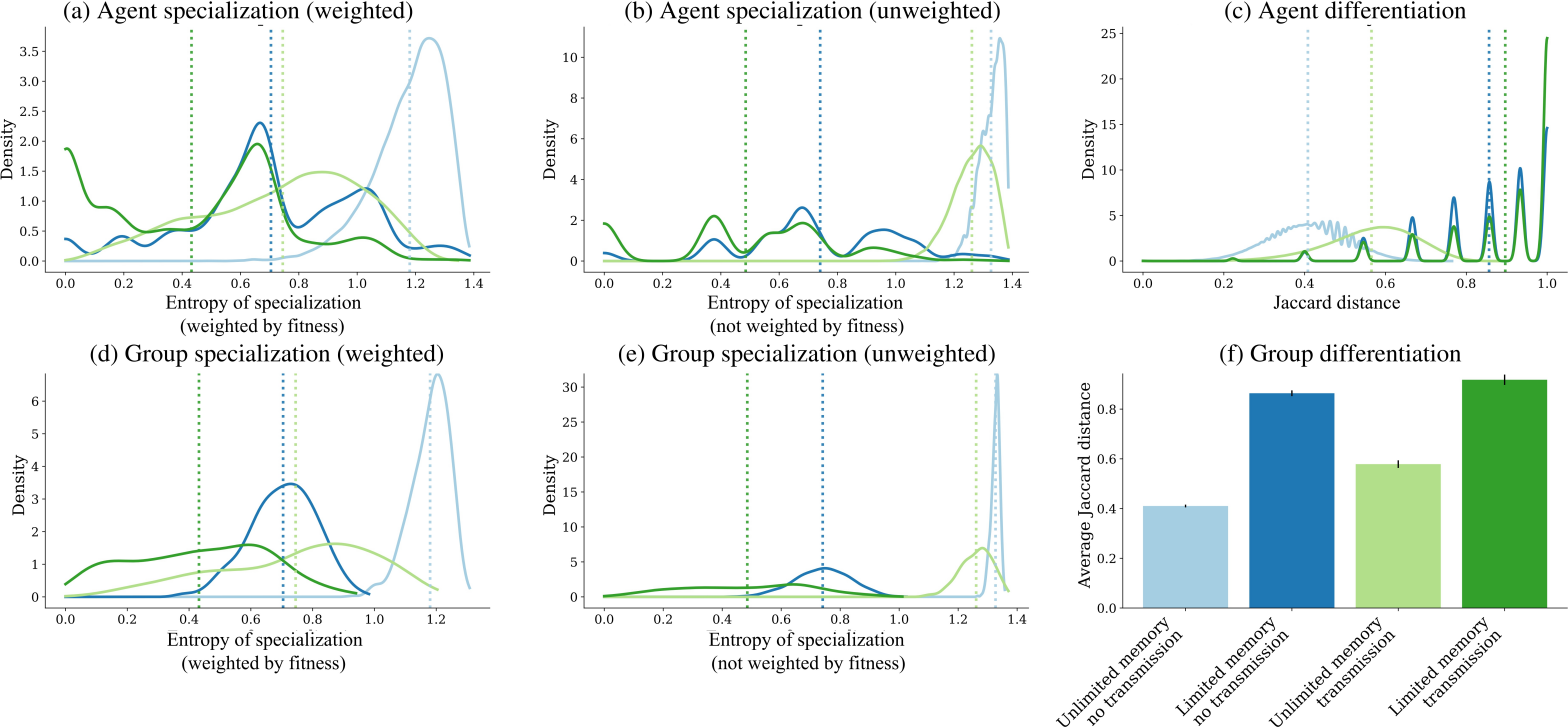
Comparison of specialization (information entropy) and differentiation (Jaccard distance) in four scenarios. (*a,b*) Individual entropies, respectively weighted and unweighted by tool fitness value; (*c*) Jaccard distances between individual agents. (*d,e*) Group entropies, weighted and unweighted by tool fitness value; (*f*) Jaccard distances between groups. In (*a–e*), dashed lines represent mean values for each scenario. Error bars in (*f*) are standard errors multiplied by 5 for better visualization. See (*f*) for colour coding of scenarios in all panels.

At the end of simulations, the highest mean entropy was observed in the unlimited memory and no transmission scenario (both with and without fitness weighting), followed by the scenario with unlimited memory and transmission (figure 3a,b). This suggests that the lack of individual cognitive costs to accumulation of growing toolkits is a factor preventing tool specialization. In simulations where agent memory was limited, mean entropy was lower and reached the lowest level when social transmission was also present. The multiple peaks of entropy values in scenarios with limited memory can be explained by the finite combinations of eight tools from the four lineages. This is more evident in the case of limited memory and social transmission, where similarity in toolkits is further enhanced by transmission to other agents. In summary, the scenario where tool evolution occurs more quickly also exhibits the highest levels of specialization and concentration of tools into fewer cultural lineages.

A related question is whether specialized agents were also differentiating their toolkits among themselves. As discussed earlier, agents across the whole network might in principle specialize in the same lineage, thus reducing overall cultural diversity. To measure levels of differentiation, we calculated Jaccard distances among all individuals in the population. Notice that Jaccard distances are expected to be highest when agents specialize in different lineages, compared with a case where agents are either generalists or specialize in tools from the same lineage. The distribution of Jaccard distances within each scenario shows that agent differentiation is highest under the conditions of memory limitation and social transmission, and lowest when memories are unlimited and there is no social transmission (figure 3c). This means that limiting agent memories has also a strong and positive effect on differentiation. Therefore, technological diversity is highest at population level when memories are limited and social transmission takes place, even though agents only master the production of a small number of tools.

### (c) Emergence of group specialization and differentiation

Next, we asked whether cultural evolution is associated with specialization and differentiation also at the group level. To address group specialization, we estimated mean agent entropy within each of the 15 groups across the four scenarios. Results confirm that limited memories and social transmission are also associated with the lowest toolkit entropies at the group level (figure 3d,e). As reported above, when fitness values of tools are taken as weights, the effect of memory limitation on specialization is more pronounced.

The following step assessed group differentiation or whether groups specialize in the same lineage (leading to overall loss of tool diversity) or different tool lineages. We aggregated the 20 agent toolkits from each group into 15 virtual group toolkits and then estimated Jaccard distances between the groups. This showed that differentiation is higher in the two scenarios with memory limitation (figure 3f). The result is not trivial, since agents with limited memories could have instead specialized in the same lineages in the different groups. Higher differentiation occurred both in the scenarios with and in the scenarios without social transmission, even though they differ in their final number of tools. In summary, memory limitation and social transmission seem to promote both group specialization and differentiation.

### (d) Tool evolution and social structure

**Figure 4.**
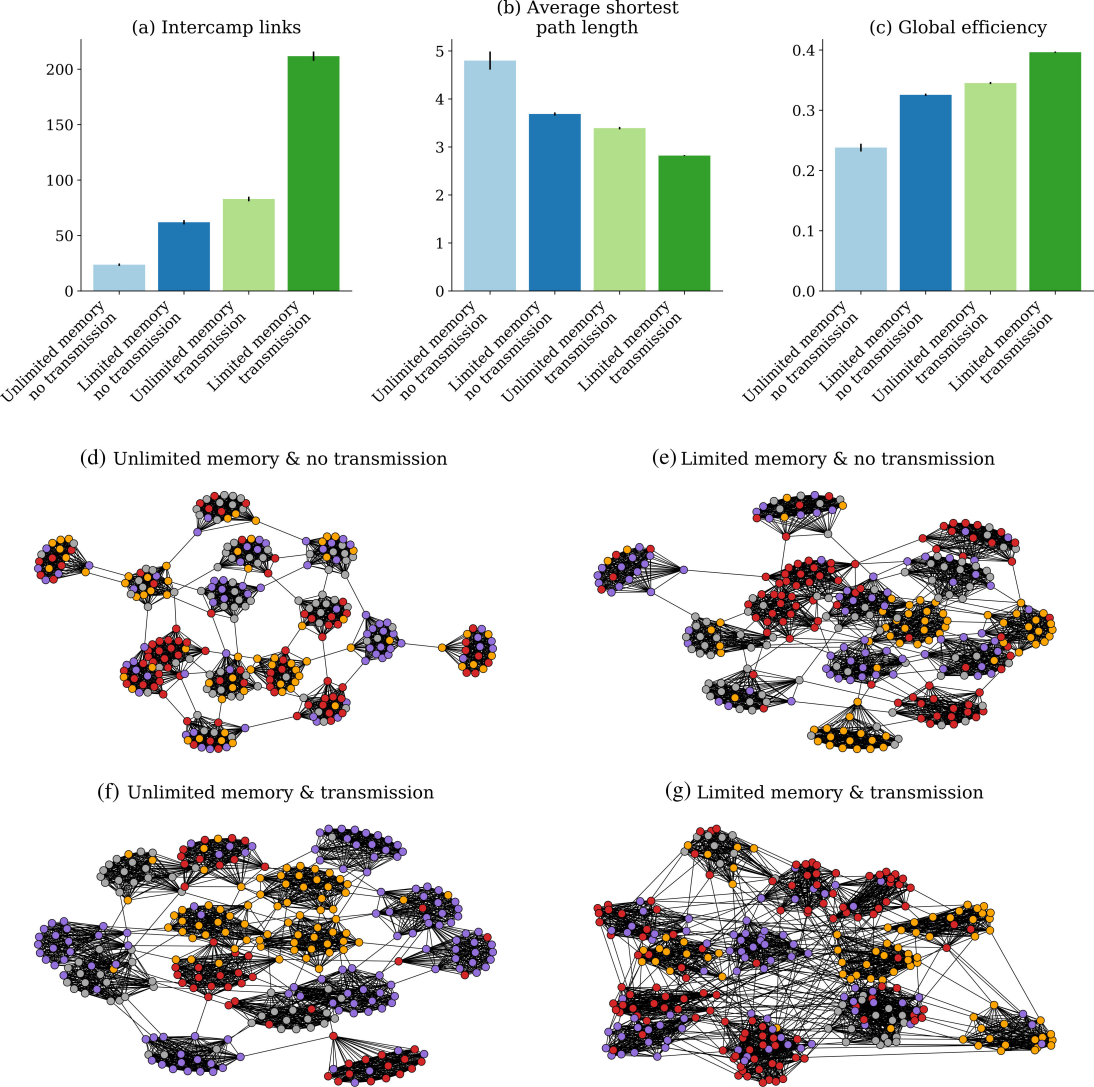
Division of labour, cultural diversity and network structure. Barplots display mean values of (*a*) number of new links between groups, (*b*) path length, and (*c*) global network efficiency in the four scenarios. Error bars represent standard errors multiplied by 5 (50 simulations each). The figure also shows a representative example of the network structure after 150 rounds of simulation under (*d*) unlimited memory, no social transmission, (*e*) limited memory, no social transmission, (*f*) unlimited memory, social transmission, and (*g*) limited memory, social transmission. Node colours represent the technological lineage of each agent at the end of the simulation.

We also investigated how selection for tools of higher values might affect link creation among groups and overall network structure. At the start of our simulations, the 15 groups were disconnected from each other. However, links between groups could be created: the simulations assumed that the probability of selection of a recombination partner from another group increased when agents from other groups carried relatively more diverse toolkits. We tracked the creation of between-group links under the four scenarios and observed that simulations with limited memory and social transmission exhibited by far the highest level of group interconnectivity, with the number of between-group links outnumbering other scenarios by a factor of at least 5 (figure 4a). The larger number of interconnections reduced path length (the average number of minimum steps between agents in a network) in the scenario with limited memory and social transmission (figure 4b), implying that the circulation of newly created tools across the whole network occurred on average through fewer steps. Overall, the effect of increased group interconnectivity on the scenario with limited memory and social transmission was a higher value of global efficiency (figure 4c), a metric that quantifies the extent to which a given network structure favours exchanges across nodes [55]. The least interconnected and globally efficient networks were created when agents had unlimited memories and did not socially transmit findings. Together, the results associate memory limitation and social transmission with the emergence of more interconnected networks (figure 4d).

### (e) Division of labour and interdependence

**Figure 5.**
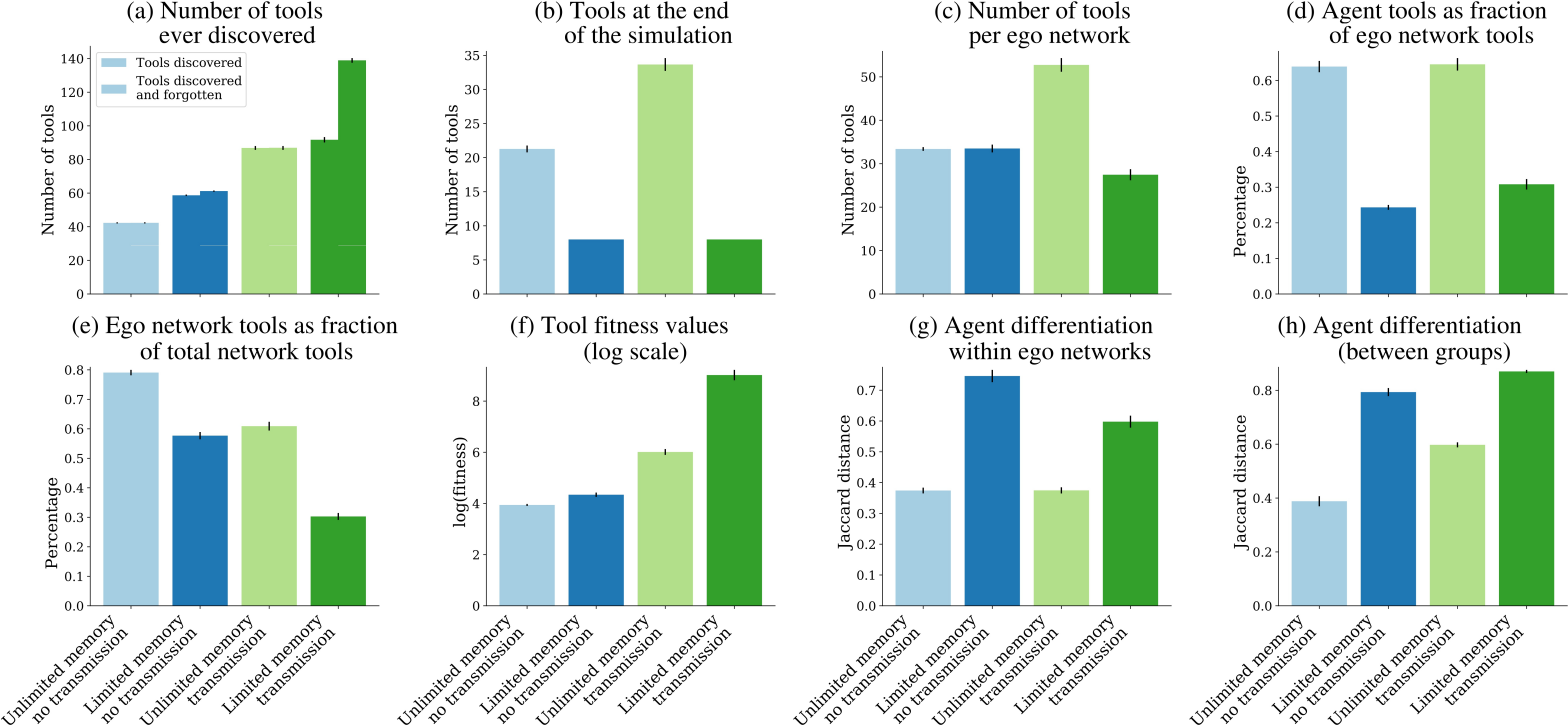
Division of labour and interdependence across four simulation scenarios. Mean values of (*a*) number of tools ever discovered (created) across 150 rounds of simulations, including both tools found in toolkits at the end of simulations, and tools created but later forgotten (replaced by others in the scenarios with limited memories); (*b*) number of tools at the end of simulations; (*c*) tools per ego network; (*d*) ego tools as fraction of ego network tools; (*e*) ego network tools as fraction of total network tools; (*f*) log of tool fitness values per agent; (*g*) Jaccard distances between toolkits within ego networks; (*h*) mean Jaccard distances between toolkits only considering the newly created between-group links. Error bars represent standard errors times 5 (from 50 simulations).

Finally, we wanted to assess whether tool evolution, specialization and differentiation were associated with division of labour and interdependence among producers. As argued above, role differentiation is not enough to demonstrate division of labour, since it is compatible with independent strategies, while under division of labour the differentiated roles evolve owing to their interdependence. Furthermore, our simulations only address division of labour in production and not in distribution of products. Therefore, we assessed whether producers that specialize in one tool lineage also interact more closely with producers of other tool lineages. We observed that agents with limited memory and social transmission create a much larger number of tools over 150 rounds (figure 5a), resulting in a larger toolkit at the population level. This occurs even though individual agents must discard their less valuable tools (forgotten tools) when a high-value tool is created and added to the toolkit. While by definition agents with infinite memory can accumulate more tools (figure 5b), an unexpected result is that the total number of tools within ego networks (consisting of an agent and all its network neighbours) is also smallest under the conditions of memory limitation and social transmission (figure 5c). Since this is the scenario where most tools are created, it means that on average these producers have direct access to a smaller fraction of the population pool required for incremental steps or crossovers. Confirming the inference, we first observed that agents with limited memory (both with and without social transmission) kept a relatively small fraction of the tools found in their own focal networks (figure 5d).

Second, the fraction of tools kept by ego networks relative to the population pool was much smaller in the scenario with limited memory, but now the scenario with social transmission was a clear outlier (under 25%), compared with all other scenarios, where the fraction was over 50% (figure 5e). Since this scenario is also where mean tool value is the highest (figure 5f), a possibility is that agents end up in ego networks with the highest levels of toolkit diversity, thereby increasing the probability of innovation. A comparison of mean Jaccard distances within ego networks confirms this interpretation, showing that the two scenarios with limited memories show the highest levels of differentiation (figure 5g). The same is observed considering only the new between-group links established during the simulations (figure 5h), which are also created in larger numbers when agents have limited memories (see figure 4a above).

## 4. Discussion

Division of labour is found in various social species. Characteristic features of human division of labour are the smaller role played by kinship in reducing within-group conflict, interdependence in production and distribution among unrelated individuals, and above all, the fundamental role that cultural evolution and transmission have played in determining individual specialization and differentiation. While it is widely assumed that cultural evolution may result in cultural ratcheting, our purpose was to provide evidence that cultural evolution will also generate social ratcheting, the growing division of labour and interdependence among specialized producers to a point where they can no longer produce or innovate their own technologies in isolation. This argument draws a clear distinction between human division of labour and instances of ecological specialization or social differentiation. The fact that such distinction is not always made may explain, for example, why our simulations show a direct association between specialization, cultural diversity and network interconnectivity, while previous studies [9] had reported an inverse relationship between specialization and cultural diversity. More studies of human division of labour explicitly considering its relation to cultural ratcheting and evolution are required.

We have also directly addressed the relation between cumulative cultural evolution and individual cognition. Often the cultural ratcheting argument is phrased in terms of the limited ability of individuals to reinvent from scratch certain traits whose creation required generations of individuals and various innovation steps. However, cumulative culture poses an equal challenge to aspects of individual cognition other than innovation, namely memory and even learning itself. In fact, humans are not only unable to reinvent various cultural traits from scratch but also unable to acquire the vast majority of human culture even when assisted by teaching and social learning. One of our main results was the demonstration that limited memories accelerate rather than hinder cultural accumulation, revealing a trade-off or inverse relationship between memory size and tool complexity. This can be explained by the role played by social networks in distributing the burden of storage and creation of cultural items. The result highlights the fact that human division of labour also takes place at the cognitive level, with individuals learning only a fraction of the totality of culture.

Finally, we mentioned that human division of labour refers to at least two interconnected dimensions: distribution and production, which may require distinct patterns of cooperation. Our study focused on interdependence only regarding the transmission of tools and skills in the context of production. Such production interactions may involve a limited number of specialists rather than the whole population. To model division of labour more generally in human societies and investigate interdependence also at the level of distribution of products, other models addressing food sharing, gift networks and trade are required. In summary, our simulations show that cultural evolution is faster when individual memories are limited and promotes cooperation at the production level among partners with more differentiated toolkits. We conclude that cultural and social ratcheting may have evolved as two outcomes of the same processes of cultural evolution in hominins.

## Data Availability

Supplementary material with all codes and simulation data is available online from Github at [56] and Figshare at [57].

## References

[1] Tomasello M . 2009 The cultural origins of human cognition. Cambridge, MA: Harvard University Press. (10.2307/j.ctvjsf4jc)

[2] Dean LG , Vale GL , Laland KN , Flynn E , Kendal RL . 2014 Human cumulative culture: a comparative perspective. Biol. Rev. **89** , 284–301. (10.1111/brv.12053) 24033987

[3] Whiten A . 2023 Cultural evolution in the science of culture and cultural evolution. Phys. Life Rev. **45** , 31–51. (10.1016/j.plrev.2023.03.001) 37003251

[4] Tennie C , Call J , Tomasello M . 2009 Ratcheting up the ratchet: on the evolution of cumulative culture. Phil. Trans. R. Soc. B **364** , 2405–2415. (10.1098/rstb.2009.0052) 19620111 PMC2865079

[5] Nakahashi W , Feldman MW . 2014 Evolution of division of labor: emergence of different activities among group members. J. Theor. Biol. **348** , 65–79. (10.1016/j.jtbi.2014.01.027) 24486228

[6] Migliano A , Vinicius L . 2022 The origins of human cumulative culture: from the foraging niche to collective intelligence. Phil. Trans. R. Soc. B **377** , 20200317. (10.1098/rstb.2020.0317) 34894737 PMC8666907

[7] Henrich J , Boyd R . 2008 Division of labor, economic specialization, and the evolution of social stratification. Curr. Anthropol. **49** , 715–724. (10.1086/587889)

[8] Migliano AB , Vinicius L . 2024 Hunter-gatherer sociality and the origin of human normative thinking. In Institutional dynamics and organizational complexity: how social rules have shaped the evolution of human societies throughout human history (eds JJW Richerson , J Bednar ). Cultural Evolution Society. (10.32942/X2R026)

[9] Smolla M , Akçay E . 2019 Cultural selection shapes network structure. Sci. Adv. **5** , eaaw0609. (10.1126/sciadv.aaw0609) 31453324 PMC6693906

[10] Forister ML , Dyer LA , Singer MS , Stireman III JO 3rd , Lill JT . 2012 Revisiting the evolution of ecological specialization, with emphasis on insect-plant interactions. Ecology **93** , 981–991. (10.1890/11-0650.1) 22764485

[11] Nowak MA , Tarnita CE , Wilson EO . 2010 The evolution of eusociality. Nature **466** , 1057–1062. (10.1038/nature09205) 20740005 PMC3279739

[12] Thorne BL . 1997 Evolution of eusociality in termites. Annu. Rev. Ecol. Syst. **28** , 27–54. (10.1146/annurev.ecolsys.28.1.27)

[13] Queller DC , Strassmann JE . 1998 Kin selection and social insects: social insects provide the most surprising predictions and satisfying tests of kin selection. Bioscience **48** , 165–175. (10.2307/1313262)

[14] Hill KR *et al* . 2011 Co-residence patterns in hunter-gatherer societies show unique human social structure. Science **331** , 1286–1289. (10.1126/science.1199071) 21393537

[15] Dyble M , Salali GD , Chaudhary N , Page A , Smith D , Thompson J , Vinicius L , Mace R , Migliano AB . 2015 Sex equality can explain the unique social structure of hunter-gatherer bands. Science **348** , 796–798. (10.1126/science.aaa5139) 25977551

[16] Whiten A , Goodall J , McGrew WC , Nishida T , Reynolds V , Sugiyama Y , Tutin CEG , Wrangham RW , Boesch C . 1999 Cultures in chimpanzees. Nature **399** , 682–685. (10.1038/21415) 10385119

[17] van Schaik CP , Ancrenaz M , Borgen G , Galdikas B , Knott CD , Singleton I , Suzuki A , Utami SS , Merrill M . 2003 Orangutan cultures and the evolution of material culture. Science **299** , 102–105. (10.1126/science.1078004) 12511649

[18] McGrew W , Ham R , White L , Tutin C , Fernandez M . 1997 Why don’t chimpanzees in Gabon crack nuts? Int. J. Primatol. **18** , 353–374. (10.1023/A:1026382316131)

[19] Wrangham RW . 2006 Chimpanzees: the culture-zone concept becomes untidy. Curr. Biol. **16** , R634–R635. (10.1016/j.cub.2006.07.031) 16920609

[20] Hobaiter C , Poisot T , Zuberbühler K , Hoppitt W , Gruber T . 2014 Social network analysis shows direct evidence for social transmission of tool use in wild chimpanzees. PLoS Biol. **12** , e1001960. (10.1371/journal.pbio.1001960) 25268798 PMC4181963

[21] Koops K , Soumah AG , van Leeuwen KL , Camara HD , Matsuzawa T . 2022 Field experiments find no evidence that chimpanzee nut cracking can be independently innovated. Nat. Hum. Behav. **6** , 487–494. (10.1038/s41562-021-01272-9) 35075258

[22] Boesch C . 2012 Wild cultures: a comparison between chimpanzee and human cultures. Cambridge, UK: Cambridge University Press.

[23] Claidière N , Messer EJE , Hoppitt W , Whiten A . 2013 Diffusion dynamics of socially learned foraging techniques in squirrel monkeys. Curr. Biol. **23** , 1251–1255. (10.1016/j.cub.2013.05.036) 23810529

[24] Gunasekaram C . 2023 Population interconnectivity shapes the distribution and complexity of chimpanzee cumulative culture. bioRxiv 2023.08.14.553272. (10.1101/2023.08.14.553272) 39571020

[25] Berdugo S , Cohen E , Davis AJ , Matsuzawa T , Carvalho S . 2024 Stable long-term individual variation in chimpanzee technological efficiency. bioRxiv 2023.11.21.568000. (10.1101/2023.11.21.568000) PMC11936830 39715870

[26] O’Malley RC , Wallauer W , Murray CM , Goodall J . 2012 The appearance and spread of ant fishing among the Kasekela chimpanzees of Gombe. Curr. Anthropol. **53** , 650–663. (10.1086/666943) 25242820 PMC4166518

[27] Sakura O , Matsuzawa T . 1991 Flexibility of wild chimpanzee nut‐cracking behavior using stone hammers and anvils: an experimental analysis. Ethology **87** , 237–248. (10.1111/j.1439-0310.1991.tb00249.x)

[28] Lewis J . 2019 Sharing pleasures to share rare things: hunter-gatherers’ dual distribution systems in Africa. In Towards a broader view of hunter-gatherer sharing (eds N Lavi , DE Friesem ), pp. 99–112. Cambridge, UK: McDonald Institute for Archaeological Research.

[29] Bombjaková D . 2018 The role of public speaking, ridicule, and play in cultural transmission among Mbendjele Bayaka Forest hunter-gatherers. PhD thesis, University College London.

[30] Stewart BA , Zhao Y , Mitchell PJ , Dewar G , Gleason JD , Blum JD . 2020 Ostrich eggshell bead strontium isotopes reveal persistent macroscale social networking across late Quaternary southern Africa. Proc. Natl Acad. Sci. USA **117** , 6453–6462. (10.1073/pnas.1921037117) 32152113 PMC7104358

[31] Wiessner P . 1998 On network analysis: the potential for understanding (and misunderstanding) !Kung Hxaro. Curr. Anthropol. **39** , 514–517. (10.1086/204763)

[32] Boomsma JJ , Beekman M , Cornwallis CK , Griffin AS , Holman L , Hughes WOH , Keller L , Oldroyd BP , Ratnieks FLW . 2011 Only full-sibling families evolved eusociality. Nature **471** , E4–E5. (10.1038/nature09832) 21430722

[33] Sagan L . 1967 On the origin of mitosing cells. J. Theor. Biol. **14** , 225–274. (10.1016/0022-5193(67)90079-3) 11541392

[34] Barton NH , Charlesworth B . 1998 Why sex and recombination? Science **281** , 1986–1990. (10.1126/science.281.5385.1986) 9748151

[35] Haldane JBS , Huxley J . 1927 Animal biology. Oxford, UK: Oxford University Press.

[36] Szathmáry E , Maynard Smith J . 1995 The major evolutionary transitions. Nature **374** , 227–232. (10.1038/374227a0) 7885442

[37] Maynard Smith J , Szathmáry E . 1997 The major transitions in evolution. Oxford, UK: Oxford University Press.

[38] Boyd R , Richerson PJ , Henrich J . 2011 The cultural niche: why social learning is essential for human adaptation. Proc. Natl Acad. Sci. USA **108** , 10918–10925. (10.1073/pnas.1100290108) 21690340 PMC3131818

[39] Antón SC , Potts R , Aiello LC . 2014 Evolution of early Homo : an integrated biological perspective. Science **345** , 1236828. (10.1126/science.1236828) 24994657

[40] Cavalli-Sforza LL , Feldman MW . 1981 Cultural transmission and evolution: a quantitative approach. Princeton, NJ: Princeton University Press. 7300842

[41] Badcock C , Boyd R , Richerson PJ . 1988 Culture and the evolutionary process. Chicago, IL: University of Chicago Press. (10.2307/2803086)

[42] Richerson PJ , Boyd R . 2010 Why possibly language evolved. Biolinguistics **4** , 289–306. (10.5964/bioling.8793)

[43] Ambrose SH . 2001 Paleolithic technology and human evolution. Science **291** , 1748–1753. (10.1126/science.1059487) 11249821

[44] Migliano AB *et al* . 2020 Hunter-gatherer multilevel sociality accelerates cumulative cultural evolution. Sci. Adv. **6** , eaax5913. (10.1126/sciadv.aax5913) 32158935 PMC7048420

[45] Salali GD *et al* . 2016 Knowledge-sharing networks in hunter-gatherers and the evolution of cumulative culture. Curr. Biol. **26** , 2516–2521. (10.1016/j.cub.2016.07.015) 27618264

[46] Derex M , Boyd R . 2016 Partial connectivity increases cultural accumulation within groups. Proc. Natl Acad. Sci. USA **113** , 2982–2987. (10.1073/pnas.1518798113) 26929364 PMC4801235

[47] Ammar M , Fogarty L , Kandler A . 2023 Social learning and memory. Proc. Natl Acad. Sci. USA **120** , e2310033120. (10.1073/pnas.2310033120) 37549253 PMC10433305

[48] Kaplan H , Hill K , Lancaster J , Hurtado AM . 2000 A theory of human life history evolution: diet, intelligence, and longevity. Evol. Anthropol. **9** , 156–185. (10.1002/1520-6505(2000)9:43.3.co;2-z)

[49] Smith D *et al* . 2019 A friend in need is a friend indeed: need-based sharing, rather than cooperative assortment, predicts experimental resource transfers among Agta hunter-gatherers. Evol. Hum. Behav. **40** , 82–89. (10.1016/j.evolhumbehav.2018.08.004)

[50] Wiessner PW . 1977 Hxaro: a regional system of reciprocity for reducing risk among the !Kung San. PhD thesis, University of Michigan.

[51] Blegen N . 2017 The earliest long-distance obsidian transport: evidence from the ∼200 ka Middle Stone Age Sibilo School Road Site, Baringo, Kenya. J. Hum. Evol. **103** , 1–19. (10.1016/j.jhevol.2016.11.002) 28166905

[52] Derex M , Perreault C , Boyd R . 2018 Divide and conquer: intermediate levels of population fragmentation maximize cultural accumulation. Phil. Trans. R. Soc. B **373** , 20170062. (10.1098/rstb.2017.0062) 29440527 PMC5812974

[53] Cantor M , Chimento M , Smeele SQ , He P , Papageorgiou D , Aplin LM , Farine DR . 2021 Social network architecture and the tempo of cumulative cultural evolution. Proc. R. Soc. B **288** , 20203107. (10.1098/rspb.2020.3107) PMC7944107 33715438

[54] Migliano AB *et al* . 2017 Characterization of hunter-gatherer networks and implications for cumulative culture. Nat. Hum. Behav **1** , 0043. (10.1038/s41562-016-0043)

[55] Latora V , Marchiori M . 2001 Efficient behavior of small-world networks. Phys. Rev. Lett. **87** , 198701. (10.1103/physrevlett.87.198701) 11690461

[56] Vinicius L , Rizzo L , Battiston F , Migliano AB . 2025 Supplementary material from: Cultural evolution, social ratcheting and the evolution of human division of labour. Github. See https://github.com/leonardorizzo/Cumulative-cultural-evolution/. 10.1098/rstb.2023.0277 PMC11969390 40109109

[57] Vinicius L , Rizzo L , Battiston F , Migliano A . 2025 Supplementary material from: Cultural evolution, social ratcheting and the evolution of human division of labour. Figshare. (10.6084/m9.figshare.c.7669650) PMC11969390 40109109

